# The Noble Army of Martyrs

**Published:** 1855-11

**Authors:** 


### "THE NOBLE ARMY OF MARTYRS."

The following tribute to the memory of the physicians who died of the recent epidemic at Norfolk and Portsmouth, is eloquent and just. Let the memory of these devoted physicians, "as the memory of all brave men should, live":—

IN MEMORY OF

| | | | |
|---|---|---|---|
| SYLVESTER, | TRUGIEN, | GOOCH, | CRAYCROFT, |
| CONSTABLE, | PARKER, | HOWLE, | MEIRSON, |
| HALSON, | LOVETT, | GELBARDT, | HANDY, |
| COLE, | WALTERS, | SYLVESTER, JR., | BLOW, |
| HIGGINS, | THOMPSON, | JACKSON, | MORSE, |
| BRIGGS, | FLIESS, | DE BERANE, | RIZER, |
| UPSHUR, | BOOTH, | OBERMULLER, | SMITH, |
| TUNSTALL, | HOWE, | DE CAPRY, | MARSHALL, |
| SELDEN, | BACHE, | HUNTER, | CRAVEN, |
| BURNS, | DILLARD, | SCHELL, | BERRY. |

At the close of a long and bloody battle, it is the custom to present a list of the killed and wounded; that sad record of the lamented dead, who have gone down to the grave midst the smoke of the conflict; that glorious record of the heroic dead, whose gallant deeds are painted on the pages of history, whose names are cherished in all hearts.

We, too, have now to tell of like men with these; of some who have fallen at the post of duty; of others who have died while serving as volunteers in a deadly campaign. With no hope of victory, with no pomp and circumstance of war to animate the heart, our brethren in Norfolk and Portsmouth have calmly, firmly discharged their duty, and have met their fate. The slaughter is now over, and we record a mortality unprecedented in history.

Forty physicians have fallen in the hopeless contest. Exhausted with fatigues and watchings; dispirited by their want of success; pressed down with the weight of responsibility resting on them, they have sunk, easy victims to an enemy whose ravages they faithfully labored to resist. Many of these men were residents of the infected cities, and though all was consternation around them, they flinched not at that trying hour; while others from all parts of our country ardently rushed to the scene of danger, and sacrificed their lives in the vain attempt to check the fearful pestilence.

No pompous funeral accompanied our brethren to their silent graves. No music, sad and mournful, rings upon the ear. They lie quietly now, but they have not died in vain. Faithfully have they fulfilled the sacred duties of their calling, and their memories remain an imperishable legacy to the profession they have ennobled and adorned.—*Va. Med. and Sur. Jour.*

